# Multimodal Imaging Reveals Improvement of Blood Supply to an Artificial Cell Transplant Site Induced by Bioluminescent Mesenchymal Stem Cells

**DOI:** 10.1007/s11307-016-0986-1

**Published:** 2016-07-27

**Authors:** Andrea Gálisová, Eva Fábryová, Daniel Jirák, Eva Sticová, Alena Lodererová, Vít Herynek, Jan Kříž, Milan Hájek

**Affiliations:** 1 Department of RadioDiagnostic and Interventional Radiology, Institute for Clinical and Experimental Medicine, Prague, Czech Republic; 2 Institute of Biophysics and Informatics, First Faculty of Medicine, Charles University in Prague, Prague, Czech Republic; 3 Center of Experimental Medicine, Institute for Clinical and Experimental Medicine, Prague, Czech Republic; 4 Department of Clinical and Transplant Pathology, Institute for Clinical and Experimental Medicine, Prague, Czech Republic; 5 Department of Pathology, Third Faculty of Medicine, Charles University, Prague, Czech Republic; 6 Diabetes Center, Institute for Clinical and Experimental Medicine, Prague, Czech Republic

**Keywords:** Magnetic resonance imaging, Dynamic contrast-enhanced MRI, DCE, Bioluminescence, Mesenchymal stem cells, Vascularisation

## Abstract

**Purpose:**

An artificial site for cell or pancreatic islet transplantation can be created using a polymeric scaffold, even though it suffers subcutaneously from improper vascularisation. A sufficient blood supply is crucial for graft survival and function and can be enhanced by transplantation of mesenchymal stem cells (MSCs). The purpose of this study was to assess the effect of syngeneic MSCs on neoangiogenesis and cell engraftment in an artificial site by multimodal imaging.

**Procedures:**

MSCs expressing a gene for luciferase were injected into the artificial subcutaneous site 7 days after scaffold implantation. MRI experiments (anatomical and dynamic contrast-enhanced images) were performed on a 4.7-T scanner using gradient echo sequences. Bioluminescent images were acquired on an IVIS Lumina optical imager. Longitudinal examination was performed for 2 months, and one animal was monitored for 16 months.

**Results:**

We confirmed the long-term presence (lasting more than 16 months) of viable donor cells inside the scaffolds using bioluminescence imaging with an optical signal peak appearing on day 3 after MSC implantation. When compared to controls, the tissue perfusion and vessel permeability in the scaffolds were significantly improved at the site with MSCs with a maximal peak on day 9 after MSC transplantation.

**Conclusions:**

Our data suggest that the maximal signal obtained by bioluminescence and magnetic resonance imaging from an artificially created site between 3 and 9 days after MSC transplantation can predict the optimal time range for subsequent cellular or tissue transplantation, including pancreatic islets.

## Introduction

Cellular replacement therapy has become a promising therapeutic option for a variety of diseases, including type 1 diabetes mellitus. Intrahepatic implantation of donor pancreatic islets (PIs) can restore normal insulin levels and prevent hypoglycaemic episodes [1, 2], although the efficacy and longevity of the procedure are not optimal. There are various limitations to intraportal islet transplantation, *e.g.,* reduction of immediate islet engraftment as well as long-term function. Other factors include non-specific inflammation [3], liver ischaemia [4], thrombosis [5], permanent hypoxia, significant excursion of nutrients, drugs and toxins in the portal vein blood and/or technically impractical graft biopsies. Therefore, an alternative transplantation site—capable of providing an adequate vascular network and minimally invasive access without any direct contact with blood—is currently needed [6].

An artificial site created using a polymeric scaffold inserted into the greater omentum or subcutaneously has already been proposed as a suitable site for PI transplantation [7]. The advantages of subcutaneous implantation are as follows: minimally invasive surgery and the possibility to easily monitor islets. However, the subcutaneous vascular network is inadequate and needs to be enhanced for successful engraftment [8–10]. Pancreatic islets are sensitive to hypoxia, which is even emphasised in environments with high concentrations of glucose typical of a diabetic recipient. Vascularisation of artificial devices can be enhanced by growth factors, such as vascular endothelial growth factor (VEGF) released in close vicinity to implanted scaffolds [11]. Mesenchymal stem cells (MSCs) secrete various immunomodulatory, anti-inflammatory and trophic factors, including VEGF [12, 13]. It has already been shown that MSCs can enhance angiogenesis both *in vitro* [14] and *in vivo* [15, 16]. Several studies have shown improved transplantation outcomes after co-transplantation of pancreatic islets with MSCs due to improved graft revascularisation [17, 18] and suppression of immune and inflammatory responses [19, 20]. Moreover, MSCs can be isolated from the stromal vascular fraction of adipose tissue [21, 22]; therefore, they represent an available and easily reachable source for potential clinical applications. Several pre-clinical studies have illustrated the contribution of adipose-derived MSCs to neovascularisation after ischaemic damage [23, 24].

In our group’s previous work, the experiments with dynamic contrast-enhanced (DCE) magnetic resonance imaging (MRI) showed that blood supply benefits from MSC transplantation [8]. However, the long-term effect of MSCs has not been examined yet. For this purpose, we used genetically modified MSCs with expression of a luciferase gene isolated from transgenic Lewis rats [25], which could be tracked specifically over the long term by bioluminescence imaging (BLI). A luciferase reaction produces light, which is dependent on the supply of oxygen and adenosine triphosphate. Therefore, only viable cells can be visualised. BLI has previously been used for monitoring the localisation and fate of transplanted MSCs [26] and pancreatic islets [27] in experimental models. Moreover, multimodal imaging of the transplanted graft combining complementary BLI and MRI methods can elucidate the local effect of viable MSCs on the vascularisation level.

The goal of this study was to examine the long-term influence of syngeneic adipose-derived MSCs on the blood supply in subcutaneously implanted artificial devices using multimodal imaging. Data relating to the level of vascularisation (obtained by imaging methods) were compared to microvascular density analysis using immunohistology slides. Based on the obtained findings, we here propose an optimal transplantation timing schedule for pancreatic islets.

## Materials and Methods

### Animal Model

**Fig. 1.**
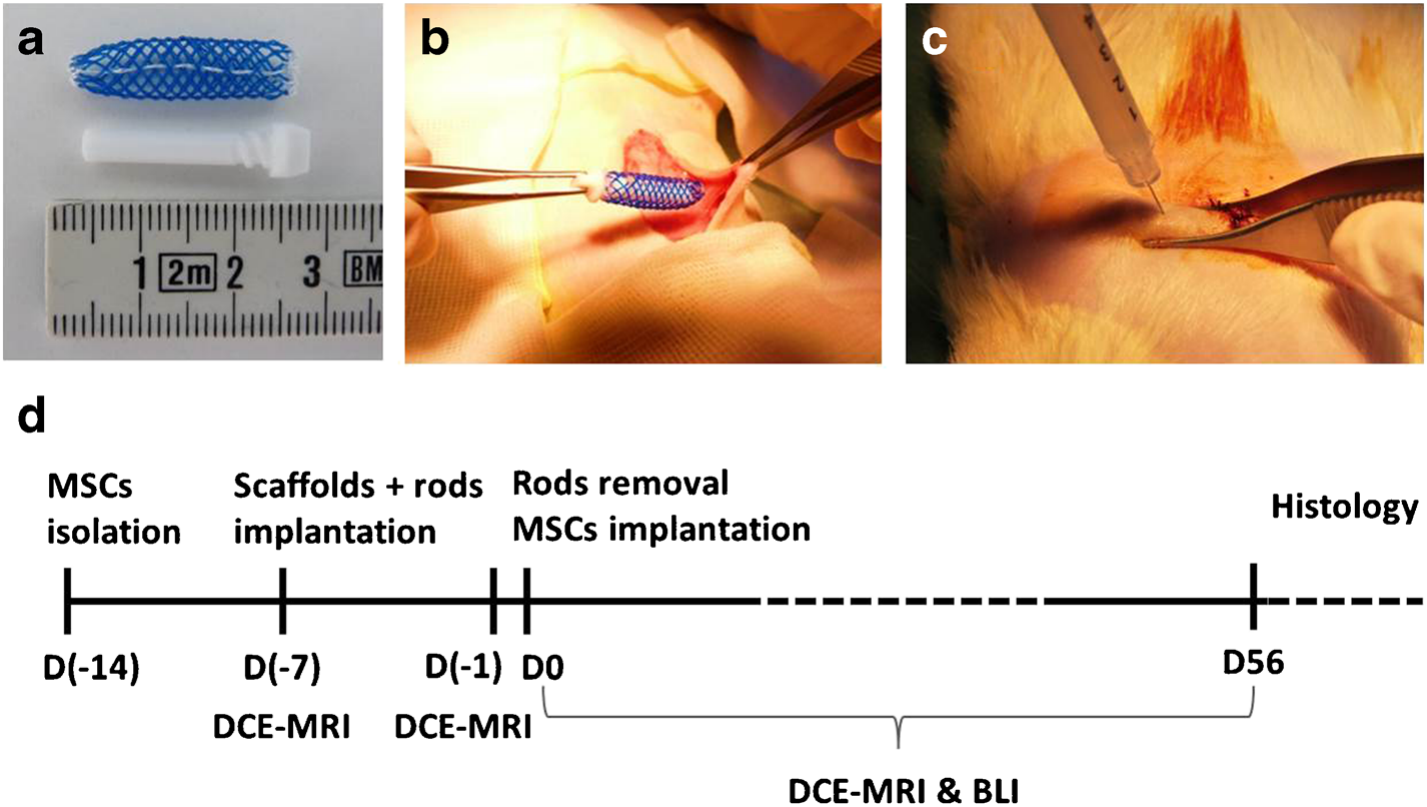
Experiment design. **a** A photograph showing a device consisting of a macroporous monofilament scaffold combined with a rounded Teflon rod. **b** A photograph showing the implantation procedure of the scaffold into the subcutaneous space in the abdominal site of the animal and injection of MSCs into the artificial site through the skin and **c** engrafted scaffold. **d** A schematic illustration of the design of the experiment.

In this study, genetically modified Lewis rats with ubiquital expression of a gene for the luciferase enzyme were used as donors of mesenchymal stem cells (Lew-Tg(Gt(ROSA)26Sor-luc)11Jmsk, National BioResource Project – Rat, Kyoto, Japan). Isolated cells were transplanted into the cavity created using a polymeric macroporous scaffold shaped from a non-degradable Silon monofilament mesh of 0.3 mm fibre diameter (ELLA-CS, Czech Republic) (Fig. 1a). Two scaffolds were implanted into the skin in the abdominal region of the male Lewis rats (250–300 g; Velaz, Czech Republic; *n* = 6) under general anaesthesia (ketamine 60 mg/kg, dexmedetomidine 0.25 mg/kg) (Fig. 1b). Total obliteration of the devices was prevented using a Teflon rod (Fig. 1a) inserted during the first week. One week after the implantation, the rods from both devices were removed and the cavities were tightly closed using 7-0 Mersilk sutures (Ethicon, Johnson & Johnson Medical Ltd., Scotland). Immediately after, 15-mil of MSCs in suspension was injected into the left cavity using a 30-G needle syringe injection (Fig. 1c). A second device served as a control without any transplanted cells. The design of the experiment is shown in Fig. 1d.

All animals were kept in a conventional breeding facility under a 12/12 light cycle regimen, with free access to pelleted food and water in accordance with the European Convention of Animal Care and the Animal Care Committee of the Institute for Clinical and Experimental Medicine. The Ministry of Health of the Czech Republic also approved the protocols related to this study.

### Stem Cell Preparation

MSCs were isolated from the visceral adipose tissue of donor rats. The fat tissue from the epididymal and perirenal area was carefully excised, washed twice with cold phosphate-buffered saline and centrifuged (500*g*, 5 min) after each wash. The rinsed tissue was digested by collagenase (Sevapharma, 1340 PZS/g, 1 mg/ml; Czech Republic) for 30 min at 37 °C. Digestion was terminated upon the addition of ice-cold fetal bovine serum (Sigma-Aldrich, USA), and the mixture was filtered through a 230-μm nylon mesh. The suspension was then centrifuged and washed three times in PBS with Euro-Collins solution (1000*g*; 10, 10 and 5 min). The tissue pellet was then overlaid with 5 ml of PBS with Euro-Collins solution and Ficoll solution (1077 g/ml, Ficoll-Paque™ Premium, GE Healthcare Bio Science AB, Sweden). The cells in the interlayer were collected, washed with PBS and seeded in a tissue culture flask (45 ml volume, 180 cm^2^ surface) with a DMEM low-glucose medium, 10 % fetal bovine serum, 5 % HEPES and 1 % l-glutamine-penicillin-streptomycin solution (Sigma-Aldrich, USA). The culture medium was replaced twice a week, and the cells were subcultured for 2 weeks after isolation. At this point, cells were released from the bottom of the culture flask using trypsin, dissolved in cold PBS, quantified and placed in the syringe before the transplantation. One aliquot from each set of MSCs was examined by fluorescent-activated cell sorting (FACS) in order to identify adherent cells. Ten thousand cells were stained with the following antibodies: anti-mouse endoglin/CD 105 (R&D Systems, USA), anti-mouse/rat CD29 (BioLegend, USA), phycoerythrin/CD44 (Abcam, UK), PE-Cy™5 Mouse Anti-Rat CD45 (BD Biosciences, USA) and anti-rat/mouse CD90.1 Thy-1.1, Thy-1.1. (E-Bioscience, USA) and were incubated for 20 min. The cells were then washed with FACS solution (PBS, 0.2 % fish skin gelatin, 0.01 % sodium azide) and analysed by flow cytometry (BD FACSCalibur, BD Biosciences, USA). The data were analysed using FlowJo 9.6.4 software (Tree Star, Inc., USA).

### Magnetic Resonance Imaging

**Fig. 2.**
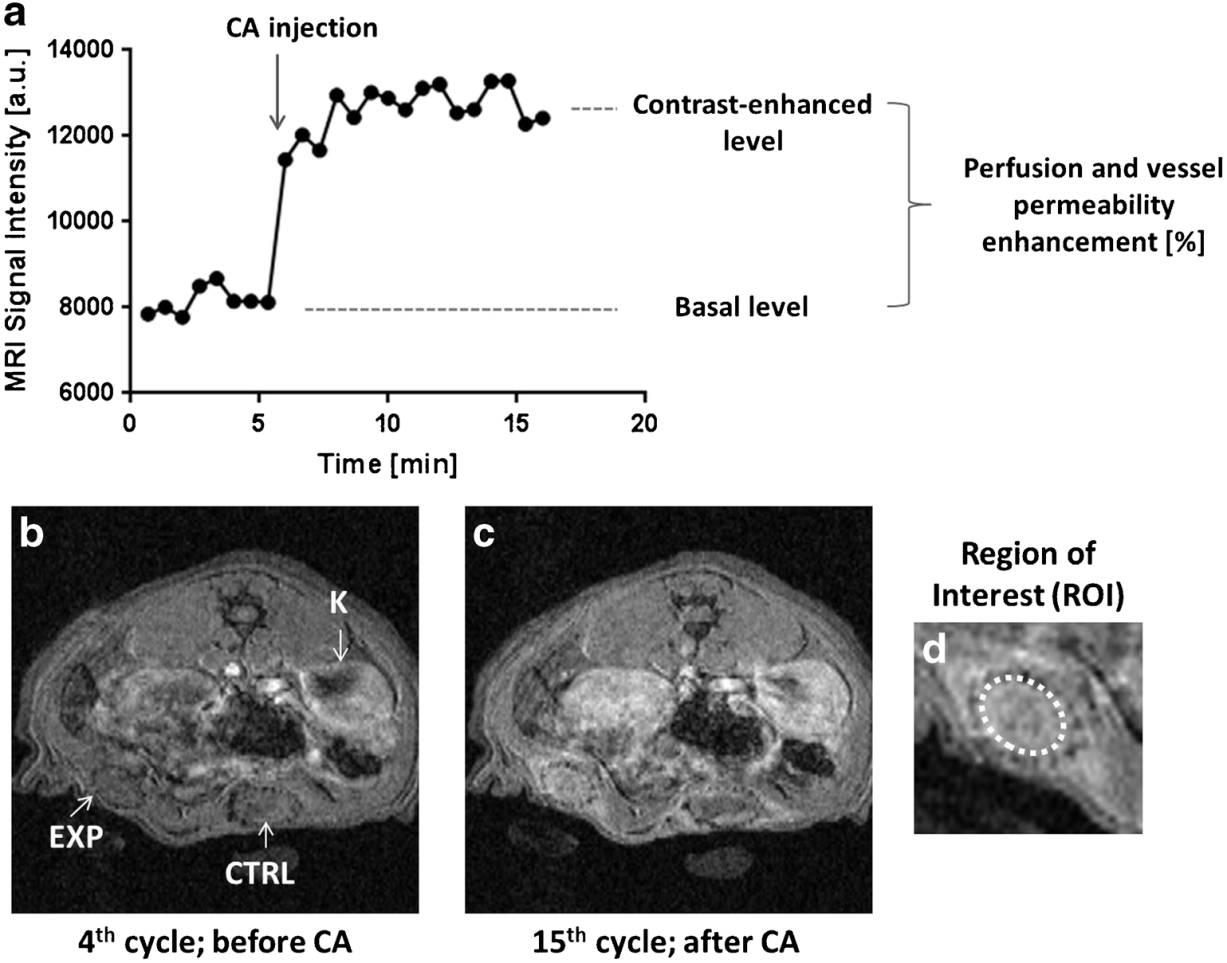
DCE-MRI measurement. **a** The graph shows MR signal intensity changes before and after contrast agent (*CA*) injection. Representative DCE-MR images **b** before and **c** after CA injection. **d** Region of interest in the artificial site chosen for evaluation. *EXP* experimental scaffold with MSCs, *CTRL* control scaffold, *K* kidney.

MRI examination was performed on a 4.7-T MR scanner (Bruker BioSpec, Germany) using a resonator coil with an internal diameter of 7 cm (Bruker BioSpec, Germany). For anatomical localisation of the devices, conventional anatomical images were acquired using a gradient echo sequence (repetition of time (TR) = 88 ms, echo time (TE) = 3.7 ms, number of acquisition (NA) = 12, acquisition time = 8 min). For dynamic measurements, a three-dimensional gradient echo sequence was used with the following parameters: TR = 10 ms, TE = 3.1 ms, matrix = 256 × 128 pixels, resolution = 0.2 × 0.4 × 0.7 mm^3^, evolution delay = 5 s, 32 slices, NA = 1, 24 cycles and acquisition time = 16 min. The temporal resolution of 1 cycle was 40 s. After the 8th cycle, the vascular-specific MR contrast agent Gadofosveset (0.05 mmol/kg) was injected into the tail vein through a catheter, avoiding the change in animal position. The data were processed using ImageJ software (version 1.46r, National Institutes of Health, USA). The slices containing both the kidney and the devices were evaluated. Regions of interest (ROIs) were outlined around the kidney and around the internal diameter of each device (Fig. 2d). The average signal intensity before injection of the contrast agent was considered as the basal level (cycles 2–8), and the mean signal intensity after administration of the contrast agent (cycles 11–24) was assessed as the contrast-enhanced level (Fig. 2a–c). The difference between basal- and contrast-enhanced signal intensity normalised to the kidney signal was calculated for every cycle of the ROI and expressed as a mean percentage enhancement. DCE-MRI experiments were performed 7 and 1 day before MSC implantation and then on days 1, 3, 5, 7, 9, 11, 14, 22, 29, 37, 42, 50 and 56 after MSC transplantation.

### Optical Imaging

Optical images were acquired on an IVIS Lumina XR imager (PerkinElmer, USA) with an exposure time of 1 min, an open aperture and an open emission filter. A photographic image was acquired for anatomical co-registration of the signal. Luciferase expression of each set of cells was confirmed using BLI. Isolated MSCs (0.6 × 10^5^ − 1 × 10^6^) were placed into a six-well plate and imaged for 1 min after the addition of 10 μl of d-luciferin (30 mg/ml). The MSC recipients were examined before and after intravenous administration of d-luciferin dissolved in sterile PBS (at a dose of 15 mg) in a time series lasting 14 min. The bioluminescent colour-coded images were superimposed on the photographic images and analysed using the Living Image software package (PerkinElmer, USA). Signal intensity was assessed as photons per second per square centimetre per steradian (p/s/cm^2^/sr) from the area containing the scaffold. The area under the curve (AUC) was calculated from each time course in order to minimise the variability in d-luciferin administration among the measurements. The bioluminescence examination was performed at the same time points as the MRI experiments; one animal was monitored for 16 months.

### Histology and Immunohistochemistry

At the end of the experiment (56 days after MSC implantation, five animals; 16 months, one animal), all transplant devices were excised from the animals, fixed overnight in 10 % buffered formalin (pH 7.4) at 4 °C and embedded in paraffin blocks. Serial sections (4 μm) were cut and stained with haematoxylin and eosin (H&E), Masson’s trichrome and elastic van Gieson stain.

Immunohistochemical detection of CD31 (rabbit polyclonal, Acris Antibodies GmbH, Germany) was performed on 4-μm-thick paraffin sections using a three-step indirect method. After deparaffinisation in xylene and rehydration in graded ethanol, antigen retrieval (EDTA buffer, pH 8), endogenous peroxidase and endogenous biotin blocking, sections were covered with normal goat serum (Vector Laboratories, USA) for 20 min. Primary antibody anti-CD31 was applied overnight at 4 °C; the antibody was detected by biotinylated goat anti-rabbit IgG (H+L) (Vector Laboratories, Burlingame, CA, USA), and then the sections were incubated with R.T.U. Vectastain Elite ABC Reagent (Vector Laboratories, USA) for 30 min. Finally, visualisation was performed with the Dako Liquid DAB+ Substrate-Chromogen System (Dako, Denmark) and counterstaining with Harris’ haematoxylin.

Each slide was viewed using standard light microscopy (Olympus BX41).

### Microvascular Density Assessment

Microvascular density (MVD) assessment was performed at the end of the 56-day experiment on two experimental and two control samples. Two paraffin blocks were prepared from each sample; three serial sections per block were stained with the primary anti-CD31 antibody. MVD was evaluated in the area of the highest vascularisation as a number of CD31-positive microvessels counted at a magnification of ×400 (*i.e.,* ×40 objective lens and ×10 ocular; 0.2375 mm^2^ per field). The results were expressed as the mean microvessel count per ×400 view field. MVD was assessed by an experienced pathologist.

### Statistical Analysis

Statistical analysis was conducted using GraphPad Prism 6.02. The average DCE-MRI signals of the control and experimental scaffolds were compared per time point using two-tailed Student’s *t* test. The significance level was set at *p* < 0.05.

## Results

### *In Vitro* Tests of MSCs

**Fig. 3.**
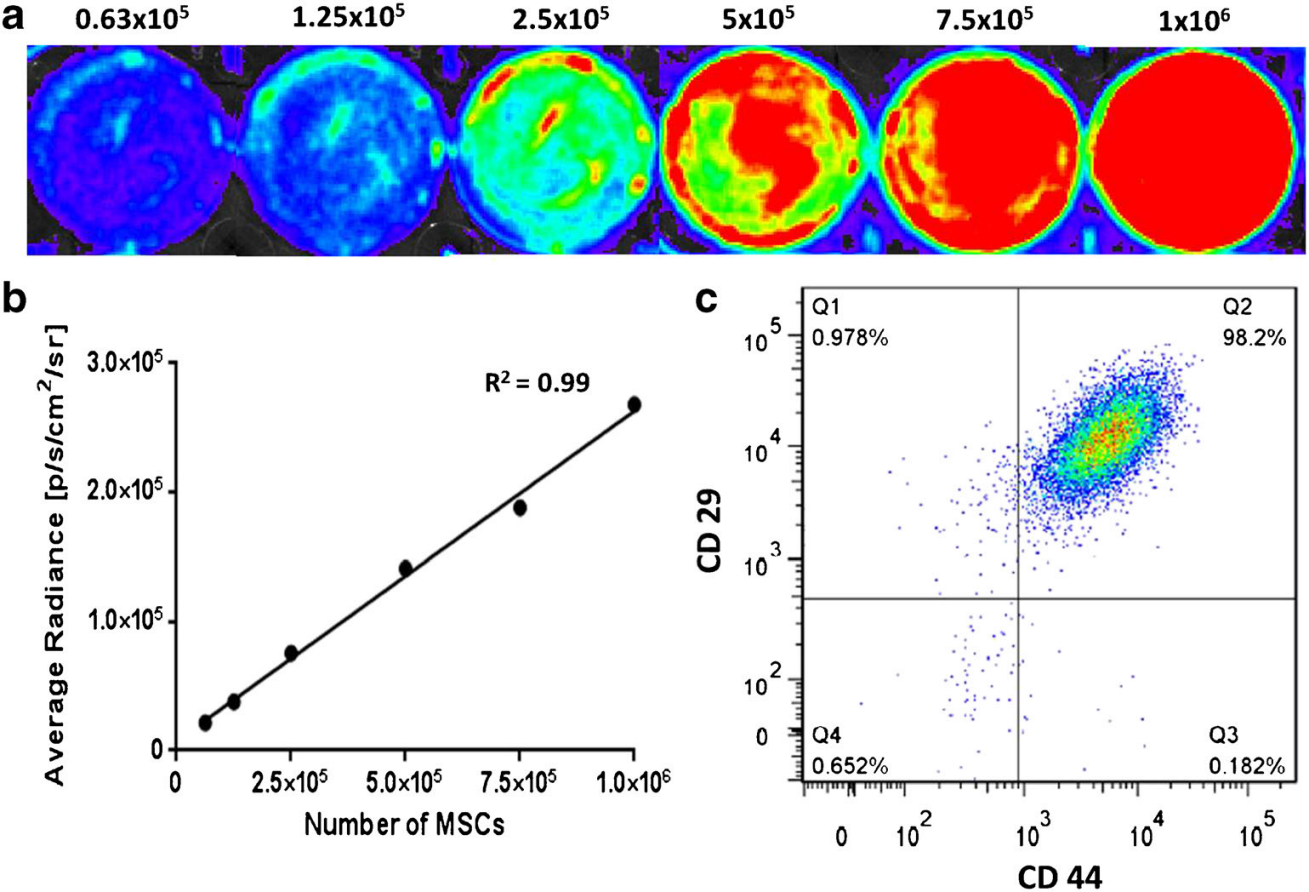
Validation of stem cell characteristics. **a** *In vitro* bioluminescent images of the isolated MSCs. **b** The linear relationship between the optical signal and cell numbers in the *in vitro* experiment. **c** The presence of CD29- and CD44-specific molecules on the MSC surface measured by FACS.

The isolated MSCs were analysed *in vitro* by flow cytometry and bioluminescence imaging to confirm their typical properties. A linear relationship between the number of the isolated MSCs and bioluminescent signal was observed (Fig. 3a, b). One million MSCs emitted 2.7 × 10^6^ photons per s from 1 cm^2^/steradian. Flow cytometry analysis revealed CD45 negativity and thus the non-haematopoietic origin of the tested cells. Moreover, the presence of the stem cell-specific molecules on the cell surfaces was confirmed: CD90 in 99.2 %, CD44 in 98.2 % and CD29 in 99.2 % of the cells (Fig. 3c).

### MRI and Bioluminescence Imaging

**Fig. 4.**
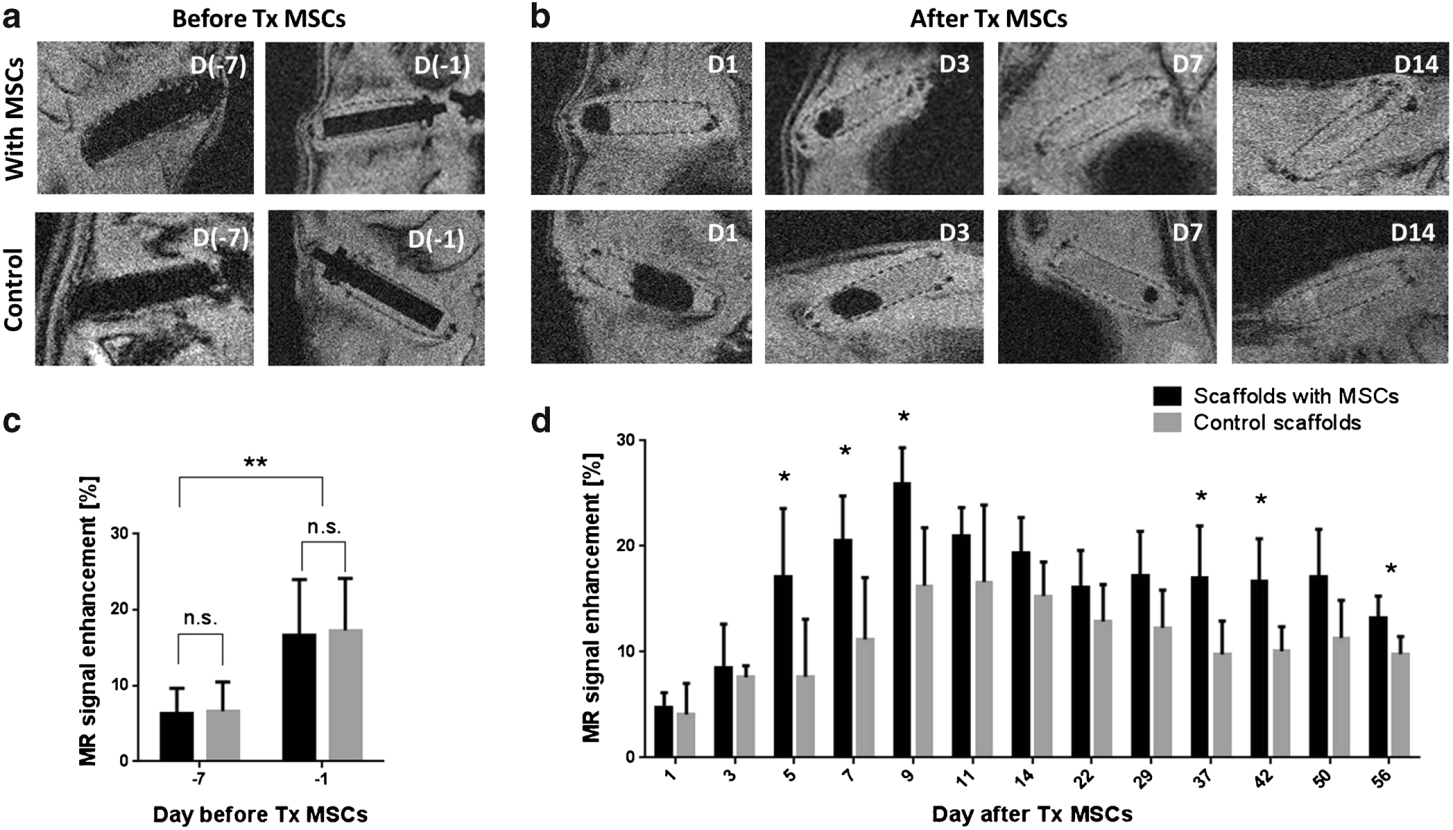
MRI examination of the scaffolds before MSC transplantation. Representative anatomical MR images of the scaffolds **a** before and **b** after MSC transplantation (*Tx*) acquired before administration of the contrast agent. MR signal enhancement related to vascularisation in the scaffolds **c** before and **d** after Tx MSCs.

MRI examination showed that tissue surrounding the Teflon rods engrafted the scaffolds within the first week (Fig. 4a). The cavities inside the devices were visualised by MRI after removal of the rod. The air bubbles were detected on days 5 and 7 after MSC transplantation, after which the cavities were filled with connective tissue (Fig. 4b). The scaffolds with MSCs were engrafted and filled with fibrous tissue faster compared to the controls. The scaffold did not cause any imaging artifacts. No difference in signal enhancement after the contrast agent administration between the two scaffolds before MSC implantation was observed, although there was a significant difference (*p* < 0.01) between days −7 and −1 (7 resp. 1 day before MSC implantation, Fig. 4c). Long-term DCE-MRI examination revealed higher signal enhancement in the devices with MSCs compared to the control devices throughout the whole experiment (Fig. 4d). The difference was significant on day 5 (*p* = 0.022), day 7 (*p* = 0.010), day 9 (*p* = 0.024), day 37 (*p* = 0.026), day 42 (*p* = 0.029) and day 56 (*p* = 0.022) after MSC implantation. The maximal significant difference was observed on day 7, when signal enhancement in the devices with MSCs reached 20.5 ± 4.3 and 11.2 ± 5.8 % in the control scaffolds. The maximal signal enhancement on day 9 was 25.9 ± 3.4 % in the scaffolds with MSCs and 16.3 ± 5.5 in the control scaffolds. At the end of the MRI experiment (day 56), perfusion and vessel permeability in both scaffolds were higher than those in the pre-transplant level with higher signal enhancement in the scaffold with MSCs (13.2 ± 2.1 %) compared to the control scaffold (9.8 ± 1.7 %).

**Fig. 5.**
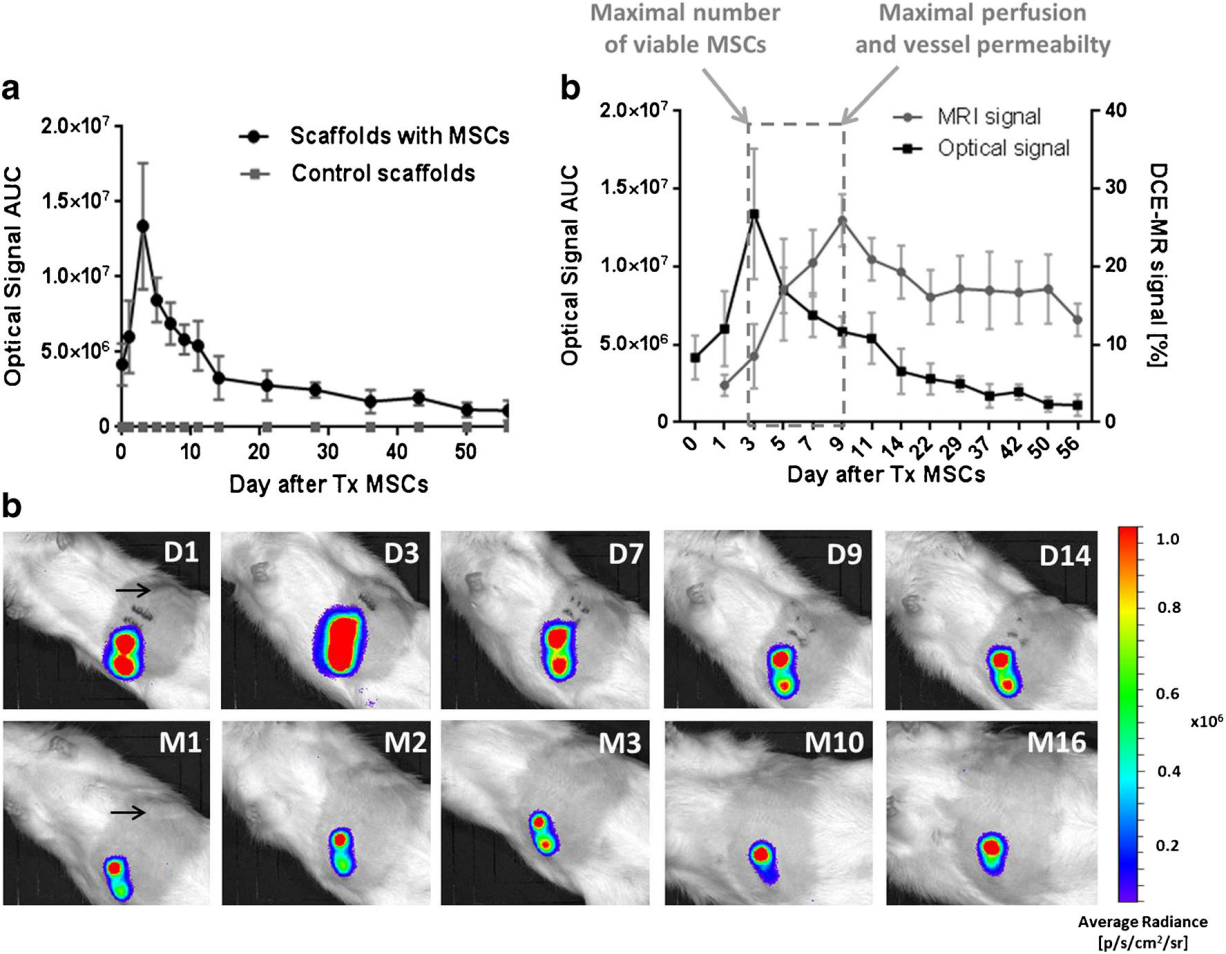
Bioluminescence imaging of transplanted MCSs in the scaffolds and the suggested transplantation window. **a** A cumulative optical signal originating from the devices at different time points after transplantation (*Tx*) of MSCs. **b** The relationship between the MRI and BLI signal at different time points after MSC transplantation. **c** Representative *in vivo* optical images of the scaffolds with bioluminescent MSCs. *Arrow* indicates control scaffold without any BLI signal. *D* day, *M* month, after MSC transplantation. **b** The suggested transplantation window between day 3 (the maximal number of viable MSCs) and day 9 (the maximal perfusion and vessel permeability) for further transplantation of pancreatic islets is expressed as a *dashed rectangle*.

Donor cells were visualised specifically inside the scaffolds by bioluminescence imaging. No bioluminescent signal was detected from the control scaffolds (Fig. 5a, c). An optical signal, confirming the presence of the viable donor cells, increased during the first 3 days after their transplantation and then continuously declined (Fig. 5a, c). A steep decrease in optical radiance was observed between days 3 and 14 after donor cell transplantation, after which the decrease slowed, resulting in a stable signal. After 2 months, the optical signal stayed at approximately 10–15 % of the maximal signal level. The stable optical signal was detected even 16 months after MSC implantation (one animal) (Fig. 5c).

In summary, the optical signal peak appeared in advance of maximal DCE-MRI signal. The optical signal reached its maximal level on day 3, after which the perfusion and vessel permeability inside the scaffolds with MSCs started to elevate rapidly before reaching the maximal level on day 9. Based on these results, we propose that the optimal transplantation window for implantation of pancreatic islet ranges between days 3 and 9, as shown in Fig. 5b.

### Histological Evaluation of the Scaffolds

**Fig. 6.**
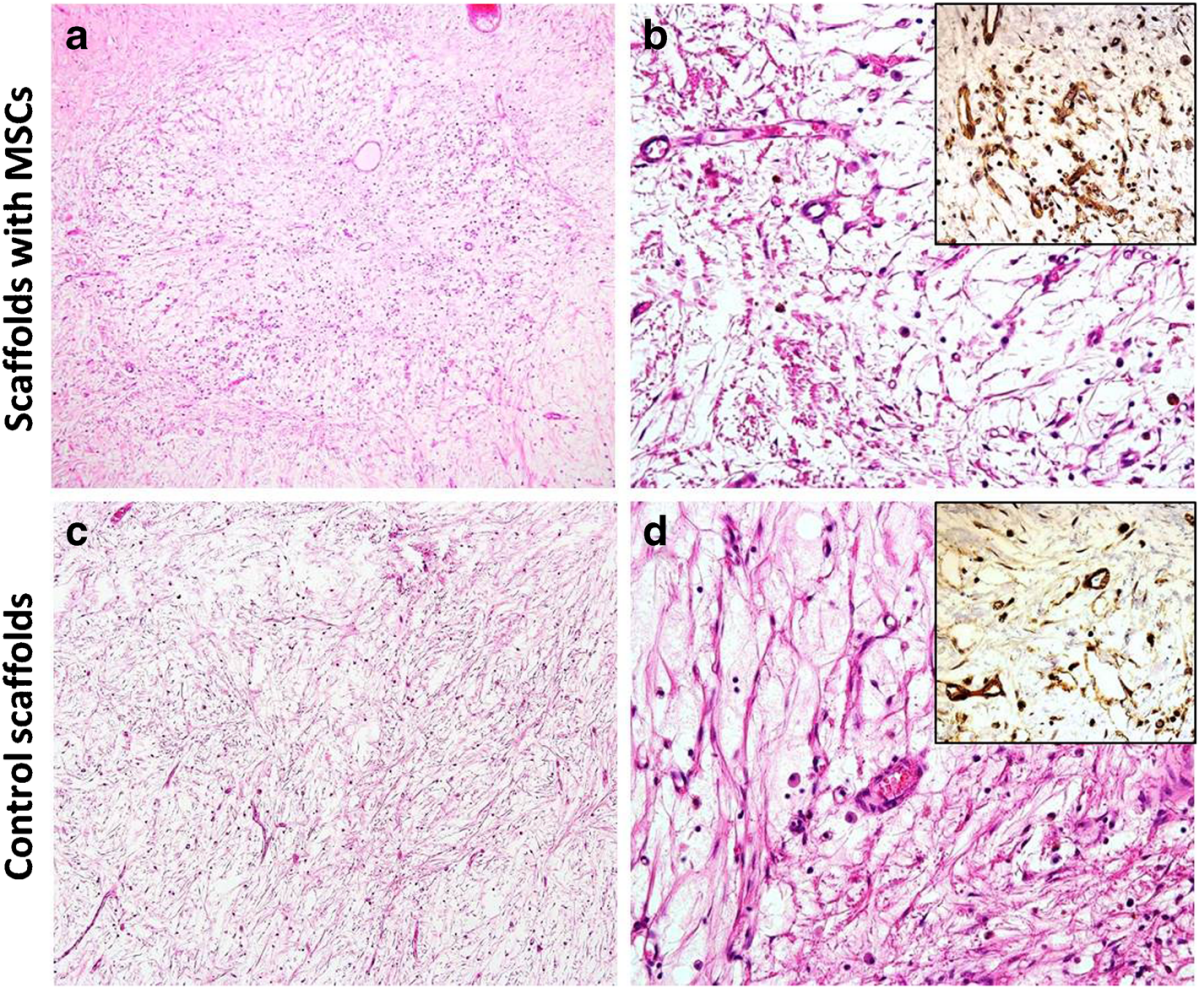
Histology of the scaffolds. The experimental (**a**, **b**) and control (**c**, **d**) scaffolds are filled with the mesenchymal tissue. Microvascular density is higher in the specimen with **b** MSCs compared to the **d** control. H&E staining (**a**–**d**), anti-CD31 immunohistochemistry (*insets*). Original magnifications ×100 (**a**, **c**) and ×400 (**b**, **d**, *insets*).

Histological analysis of the specimens showed both the experimental scaffolds containing MSCs and controls completely filled by sparse connective tissue composed predominantly of collagen, extracellular matrix and cellular component, including fibroblasts, macrophages and endothelial cells (Fig. 6a, c). The immunohistochemical staining with the anti-CD31 antibody highlighted the neovessels within the devices (Fig. 6b, d, insets). Quantitative analysis showed higher MVD in the experimental scaffolds with MSCs compared to controls: the mean microvessel count per ×400 field was 17 ± 12 (median 14; range 2 to 40) in the scaffolds with MSCs and 8 ± 3 (median 8; range 2 to 15) in controls without MSCs.

## Discussion

Non-degradable polymeric mesh can serve as a scaffold for creation of an artificial transplantation site for cells or pancreatic islets, although those implanted subcutaneously suffer from insufficient vascularisation and associated hypoxia [7]. The improvement of blood supply into scaffolds after transplantation of mesenchymal stem cells has already been demonstrated over a 4-week period, but the long-term effect has only been predicted [8]. In the present study, MSCs with luciferase expression were implemented in order to assess the effect of MSCs on scaffold engraftment in a longer period and in more detail using multimodal imaging.

MR examination during the first week after scaffold implantation revealed a cavity created by fibrous and granulation tissue, which was suitable for subsequent transplantation of MSCs. The absence of any difference in DCE-MRI signal between the experimental and control scaffolds before MCS transplantation confirmed the similar condition in both sites at day 0. One week after the insertion of MSCs, the scaffolds were fully filled by the tissue enabling vessel growth throughout the whole scaffold volume and thus providing an adequate environment for the transplanted cells. Moreover, the layer of the fibrous tissue was still thin enough to allow the oxygen to penetrate.

The survival of transplanted MSCs and/or cells differentiated from them was monitored using detection of bioluminescence signal generated by luciferase enzyme processing of d-luciferin substrate. Bioluminescent MSCs and pancreatic islets have previously been transplanted and visualised at different sites, such as the liver, kidney capsule [27], heart [28] and under skin [29]. Our results showed that bioluminescence imaging is a valuable strategy for tracking the long-term fate of transplanted MSCs and their approximate number in the artificial scaffolds. An advantage of the method is that only viable cells are capable of producing light and, therefore, being visualised [30]. However, the use of optical methods is limited by the attenuation of light signal passing though the tissues [31]. In our model, attenuation of the optical signal was minimised by subcutaneous transplantation of MSCs, which provided a robust and stable optical signal. Moreover, a linear relationship between the optical signal and the number of cells *in vitro* (Fig. 3) together with an *in vivo* optical signal steady over a period of more than 16 months post-implantation indicated a stable expression for luciferase. During the first post-transplant week, a strong optical signal was observed from the transplanted MSCs gradually increased with the peak on day 3. This reflected the maximal number of viable MSCs and confirmed the adequate availability of oxygen and the substrate d-luciferin for the luciferase reaction. Also, neoangiogenesis as well as adaptation of MSCs in the host may contribute to the gradual increase of the optical signal. The ensuing progressive decline of the bioluminescent signal during the second post-transplant week may reflect the decrease in transplanted cell mass. These observations are in accordance with previously reported gradual decrease in bioluminescence signal during the first week after transplantation of MSCs in a spinal cord injury model [32] and in transplanted pancreatic islets [33]. The reduction of isogeneic transplanted mass during the first 2 weeks was previously documented in an animal study [34, 35] and also in clinics [36]. The optical MSC signal decreased to approximately 10–15 % of initial intensity after 21 days and then remained stable until the end of the experiment (up to 16 months), confirming the long-term viability of the transplanted cells in the scaffolds and stable expression of the gene for luciferase.

The real effect of MSCs on vascular network at the transplantation site was confirmed by the dynamic contrast-enhanced MRI, which was previously reported as a valuable tool for estimation of vascularisation in a target region [9]. Changes in signal intensity after administration of the contrast agent reflected extravasation of the contrast agent, which is dependent on the number and density of the vessels. In our study, the MR signal related to tissue perfusion and vessel permeability significantly increased over a one-week period after scaffold implantation, which reflects ingrowth of the tissue together with increased vascularisation. The perfusion and vessel permeability in the region of both scaffolds had rapidly increased over the first 9 days after MSC transplantation. This increase could be related to the general reaction to the foreign body, which is accompanied by the presence of macrophages, infiltration of fibroblasts and neovascularisation in the new healing tissue [37]. This process was observed and confirmed by the histology in our model. The MSCs significantly enhanced the local perfusion and vessel permeability in comparison to controls throughout the whole examination period, which reflects the positive effect of MSCs on vascularisation, which can be caused by secretion of immunomodulatory and vascular growth factors by MSCs [12, 16]. The highest difference between the controls and scaffolds supported by MSCs was observed between days 5 and 9 after MSC transplantation. During this period, possible proliferation of MSCs may also contribute to the increased production of VEGF and thus increased neovascularisation. Enhancement of neoangiogenesis has previously been monitored in animal models [14]. Although optical imaging confirmed the long-term viability of the transplanted cells, we cannot exclude possible differentiation of MSCs into other cell types with no substantial effect on vascularisation during later stages of the experiment.

Our observation of long-term vascularisation improvement in the scaffolds containing MSCs over at least a 2-month period was confirmed histologically, as anti-CD31 staining for endothelial cells identified the markedly higher formation of new vessels in the scaffolds with MSCs. Increased microvascular density in the scaffolds with MSCs compared to controls corresponded with the results obtained by DCE-MRI. We may also speculate that the possible effect of hypoxia early after MSC transplantation may induce transcriptionally active hypoxia-inducible factors (HIFs), which enhance the expression of VEGF, platelet-derived growth factor (PDGF) and angiopoietin-2 [38, 39].

Taken together, the bioluminescence peak appearing on day 3 and the maximal perfusion and vessel permeability detected by DCE-MRI on day 9 after MSC implantation suggest an optimal time window for subsequent transplantation of pancreatic islets. Although previous studies have shown lower blood supply in the subcutaneous scaffolds compared to those implanted into the greater omentum [9], we show that engraftment of MSCs and the optimal timing could overcome this limitation.

## Conclusion

We confirmed the positive effect of adipose tissue-derived mesenchymal stem cells on vascularisation of the tissue at the wall of the artificial transplantation site via increased tissue perfusion and vessel permeability and higher microvascular density. The long-term presence of viable cells of donor origin inside the scaffolds observed by bioluminescence imaging supports this finding. Multimodal imaging revealed the optimal timing for further cell implantation of the scaffolds. Improved vascularisation, proper timing of the transplantation steps and prolongation of cell survival due to implantation of MSCs might ameliorate the efficacy of transplantation outcomes in further applications.
